# Long-term stability and reusability of molecularly imprinted polymers†
†Electronic supplementary information (ESI) available: NMR, BET and elemental analysis. See DOI: 10.1039/c6py01853j



**DOI:** 10.1039/c6py01853j

**Published:** 2016-11-24

**Authors:** Jozsef Kupai, Mayamin Razali, Sibel Buyuktiryaki, Rustem Kecili, Gyorgy Szekely

**Affiliations:** a School of Chemical Engineering & Analytical Science , The University of Manchester , The Mill , Sackville Street , Manchester , M13 9PL and UK . Email: gyorgy.szekely@manchester.ac.uk; b Department of Organic Chemistry & Technology , Budapest University of Technology & Economics , Szent Gellert ter 4 , Budapest , 1117 , Hungary; c Yunus Emre Vocational School , Anadolu University , Eskisehir , 26470 , Turkey

## Abstract

Molecularly imprinted materials are man-made mimics of biological receptors. Their polymer network has recognition sites complementary to a substrate in terms of size, shape and chemical functionality. They have diverse applications in various chemical, biomedical and engineering fields such as solid phase extraction, catalysis, drug delivery, pharmaceutical purification, (bio)sensors, water treatment, membrane separations and proteomics. The stability and reusability of molecularly imprinted polymers (IPs) have crucial roles in developing applications that are reliable, economic and sustainable. In the present article the effect of crosslinkers, functional monomers and conditions for template extraction on the long-term stability and reusability of IPs was systematically investigated. Adsorption capacity, selectivity, morphology and thermal decomposition of eleven different l-phenylalanine methyl ester imprinted polymers were studied to reveal performance loss over 100 adsorption–regeneration cycles. Furthermore, crosslinker and functional monomer specific reversible and irreversible decomposition of imprinted polymers as a result of adsorbent regeneration were investigated through adsorption studies, electron microscopy, N_2_ adsorption and thermogravimetric analysis. A decomposition mechanism was proposed and revealed using NMR spectroscopy. Solutions to avoid or overcome the limitations of the most common crosslinkers, functional monomers and extraction techniques were proposed and experimentally validated.

## Abstract 2

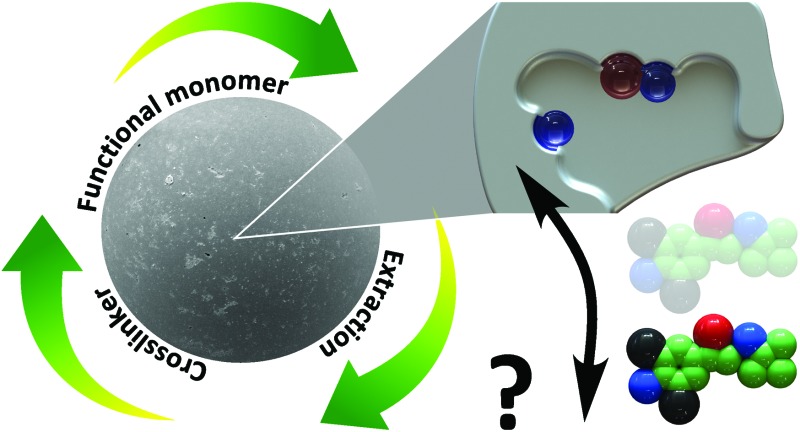
The effect of crosslinker, functional monomer and extraction on the long-term performance and degradation of molecularly imprinted polymers was investigated through adsorption studies, NMR, SEM, TGA and BET.

## Introduction

Molecular imprinting is a technique to design robust molecular recognition materials able to mimic natural recognition entities, such as antibodies and biological receptors. The preparation and application of molecularly imprinted polymers (IPs) have been thoroughly reviewed.^1^ Briefly, a specific compound is present during the polymerisation process which acts as a molecular template. Building blocks are allowed to self-assemble with the template, and the functional groups are held in position *via* crosslinking polymerisation. Subsequent removal of the template by solvent extraction or chemical cleavage leaves binding sites that are complementary to the template in terms of both topography and chemical functionality. Numerous applications of IPs have been reported such as catalysis, drug delivery and purification, sensors, water treatment, membranes and proteomics.^2–7^ Apart from the single-use analytical devices most of the chemical and engineering applications require regeneration of the IPs to improve efficiency, economics and sustainability.

The reusability of imprinted materials has a crucial role in developing applications that are reliable, economic and sustainable. Nonetheless, reusability studies in the literature are limited to about 10 adsorption cycles and there is no assessment of long-term stability and reusability of imprinted polymers. Although the monomer-template assemblies and their stability have been thoroughly studied both theoretically and experimentally,^8–12^ the sole investigation into the stability of IPs was reported by Svenson *et al.* for a theophylline imprinted methacrylate based copolymer.^13^ It was demonstrated that exposure to acids (1–10 M HCl), bases (5–25% NH_3_), autoclave treatment and elevated temperatures up to 150 °C over a period of 24 hours does not result in loss of affinity for the template. Despite its importance, the literature on template extraction itself is also scarce and intended to reduce template leaching.^14–16^

**Fig. 1:**
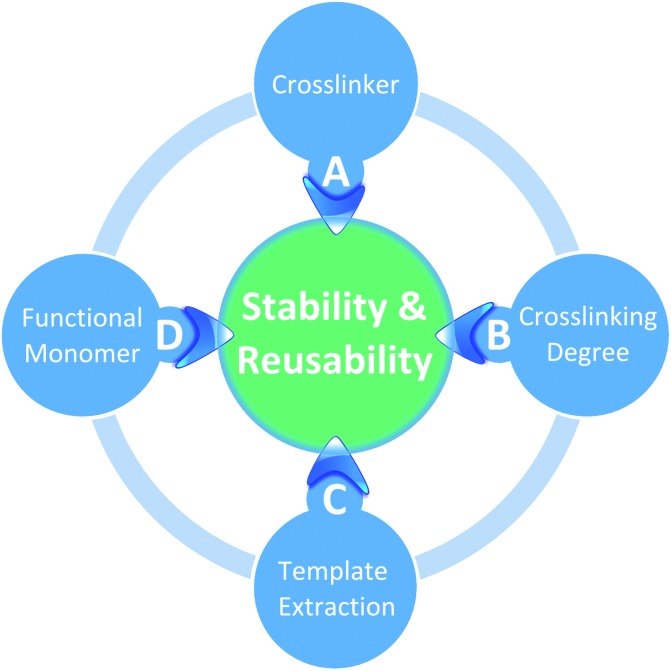
The four main factors affecting the stability and reusability of molecularly imprinted polymers.

**Fig. 2:**
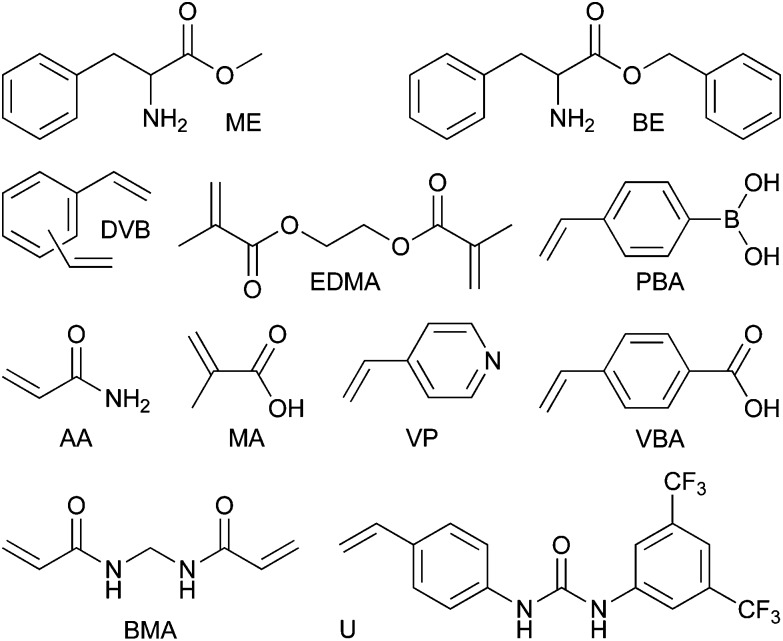
The l-phenylalanine methyl ester (ME) and benzyl ester (BE) were used as template and dummy template in this study. Ethylene glycol dimethacrylate (EDMA), divinylbenzene (DVB) and *N*,*N*′-methylenebis(acrylamide) (BMA) were employed as cross-linkers whilst methacrylic acid (MA), acrylamide (AA), 1-(4-vinylphenyl)-3-(3,5-bis(trifluoromethyl)phenyl)urea (U), 4-vinylpyridine (VP), 4-vinylphenylboronic acid (PBA) and 4-vinylbenzoic acid (VBA) were used as functional monomers.

The present study systematically investigates the effect of crosslinkers, functional monomers and conditions for template extraction on the long-term stability and reusability of imprinted polymers (Fig. 1). Twenty-four-hour adsorption–regeneration cycles for more than a 100 times were carried out in duplicate, using eleven different l-phenylalanine methyl ester (ME) imprinted polymers having different compositions. Fig. 2 shows the structures of the template, functional monomers and crosslinkers employed in the present study. Although ME has biological relevance as it is the precursor and metabolite of the synthetic sweetener aspartame,^17,18^ the application of the prepared IPs is not the focal point of the present study. ME was chosen as the template because of its multifaceted chemical nature having hydrogen donor and acceptor sites as well as an aromatic moiety capable of π–π interactions. Having such a versatile template in hand allowed investigating a wide range of functional monomers such as carboxylic acid, urea, amide, amine and boronic acid derivatives.

Acrylate, acrylamide and styrene based polymers were prepared using commonly employed crosslinkers such as ethylene glycol dimethacrylate (EDMA), divinylbenzene (DVB) and *N*,*N*′-methylenebis(acrylamide) (BMA). The crosslinker is important in controlling the morphology of the polymer matrix, serves to stabilize the imprinted binding sites, and imparts mechanical stability to the polymer matrix in order to retain its molecular recognition capability. The crosslinking degree was set at 1/15 and 1/30 functional monomer to crosslinker ratios. The template extraction was carried out with washing solvents having different eluent strength at both room (25 °C) and elevated (65 °C) temperatures. Besides fundamental understanding, the present study aims to provide guidance on adsorbent regeneration for both professionals and academicians working in the field of polymers, in particular, imprinted materials.

## Experimental section

### General

Reagents (reagent grade) and solvents (analytical grade) were purchased from Sigma-Aldrich and Fisher Scientific, respectively. The 1-(4-vinylphenyl)-3-(3,5-bis(trifluoromethyl)phenyl)urea functional monomer was synthesized following the protocol reported by Hall *et al.*,^19^ and its characterisation is included in the ESI.† Acetonitrile porogen was dried over 4 Å molecular sieves. AIBN was purchased from Wako Pure Chemical Industry and purified by recrystallization from methanol. MA and EDMA were washed with 1 M aqueous NaOH solution with distilled water, dried over anhydrous Na_2_SO_4_ and distilled over CaH_2_ under reduced pressure before use. A VWR-Hitachi Chromaster DAD-HPLC system equipped with an ACE 5 μm, C18, 300 Å, 250 × 4.6 mm column was used for template analysis (260 nm) with an isocratic elution using 25% acetonitrile and 75% phosphate buffer (pH = 7). Elemental analyses were performed in the Microanalysis Laboratory of the School of Chemistry at The University of Manchester. The nitrogen sorption measurements were performed on a Quantachrome Autosorb 6B automatic adsorption instrument. Thermal gravimetric analysis (TGA) was performed with a Seiko Instrument Inc. TG-DTA 6200 using an alumina pan under a 50 mL min^–1^ nitrogen flow at a heating rate of 10 °C min^–1^. NMR spectra were recorded in either CDCl_3_ or DMSO-*d*_6_ on a Bruker DRX-500 Avance spectrometer (at 500 MHz for ^1^H and at 125 MHz for ^13^C spectra).

### Preparation of imprinted polymers

IP microspheres were prepared by a suspension polymerisation method.^20^ Briefly, in a typical IP fabrication procedure 1 mmol functional monomer, 1 mmol template, 20 mmol crosslinker, 0.1 wt% initiator, 100 mg perfluoro polymeric surfactant (PFPS) emulsifier, 80 mL perfluoro methylcyclohexane (PMC) dispersing phase and 15 mL acetonitrile porogen were stirred at 300 rpm (Table 1). The imprinted polymers were obtained by polymerisation involving irradiation of the stirred mixture with UV light for 6 hours at a wavelength of 365 nm at room temperature under an inert atmosphere. The resulting beads were filtered and the remaining template and unreacted molecules were extracted by sequential two-hour Soxhlet extraction with methanol at 65 °C. The IPs were dried under reduced pressure for 12 h at 25 °C. Control polymers were prepared under identical conditions in the absence of a template. Different polymerisation techniques result in different morphologies that can affect the polymer degradation.

**Table 1:** Stoichiometry and preparation conditions for the ME imprinted polymers. Corresponding control polymers (CP1–CP11) were prepared in the absence of template

Polymer	Stoichiometry (mmol/mmol/mmol)	Porogen (mL)	PFPS (mg)	PMC (mL)
IP1	ME/MA/EDMA (1/1/15)	15	75	60
IP2	ME/MA/EDMA (1/1/30)	15	150	120
IP3	ME/MA/DVB (1/1/15)	15	75	60
IP4	ME/MA/DVB (1/1/30)	15	150	120
IP5	ME/MA/BMA (1/1/15)	15	75	60
IP6	ME/MA/BMA (1/1/30)	15	150	120
IP7	ME/AA/DVB (1/1/15)	15	75	60
IP8	ME/U/DVB (1/1/15)	15	75	60
IP9	ME/VP/DVB (1/1/15)	15	75	60
IP10	ME/PBA/DVB (1/1/15)	15	75	60
IP11	ME/VBA/DVB (1/1/15)	15	75	60

### Adsorption–regeneration cycles

One adsorption–regeneration cycle consisted of loading the template, reaching equilibrium adsorption, followed by the extraction of the template and drying of the polymers. For the adsorption 200 mL 1 mM template in acetonitrile was loaded per gram of adsorbent, which was then shaken at 300 rpm at 25 °C for 24 hours in an incubator. The regeneration procedures under evaluation are compared in Table 2. Either solid phase extraction (SPE) or Soxhlet extractions (SXE) were used to remove the template and regenerate the polymers.

**Table 2:** Conditions for template extraction, *i.e.* polymer regeneration. Concentrations of hydrochloric acid and sodium hydroxide for the methanolic, aqueous solutions were 0.1 M

#	Solvent	*T* (°C)	*t* (h)	Method
E1	MeOH	25	2	SPE
E2	MeOH	65	2	SXE
E3	HCl in MeOH then water	25	3	SPE
E4	HCl in MeOH then aq. HCl	25	3	SPE
E5	NaOH in MeOH then water	25	3	SPE
E6	NaOH in MeOH then aq. HCl	25	3	SPE

120 mL of 0.1 M HCl or NaOH in methanol as washing solvents per gram of adsorbent were used for extraction. Either SPE cartridges at a washing flow rate of 1 mL min^–1^ at room temperature (25 °C) or two-hours SXE at the boiling point of the solvent (65 °C) were used. The extraction was followed by a 100 mL methanol wash per gram of adsorbent at a flow rate of 1 mL min^–1^ at room temperature in order to neutralise the polymers. 0.01 mbar vacuum pressure at room temperature was applied for 12 hours in order to dry the adsorbents. The experiments were carried out in duplicate with independently prepared polymers and the resulting standard deviations are shown in the adsorption diagrams.

## Results and discussion

### Initial adsorption performance

**Fig. 3:**
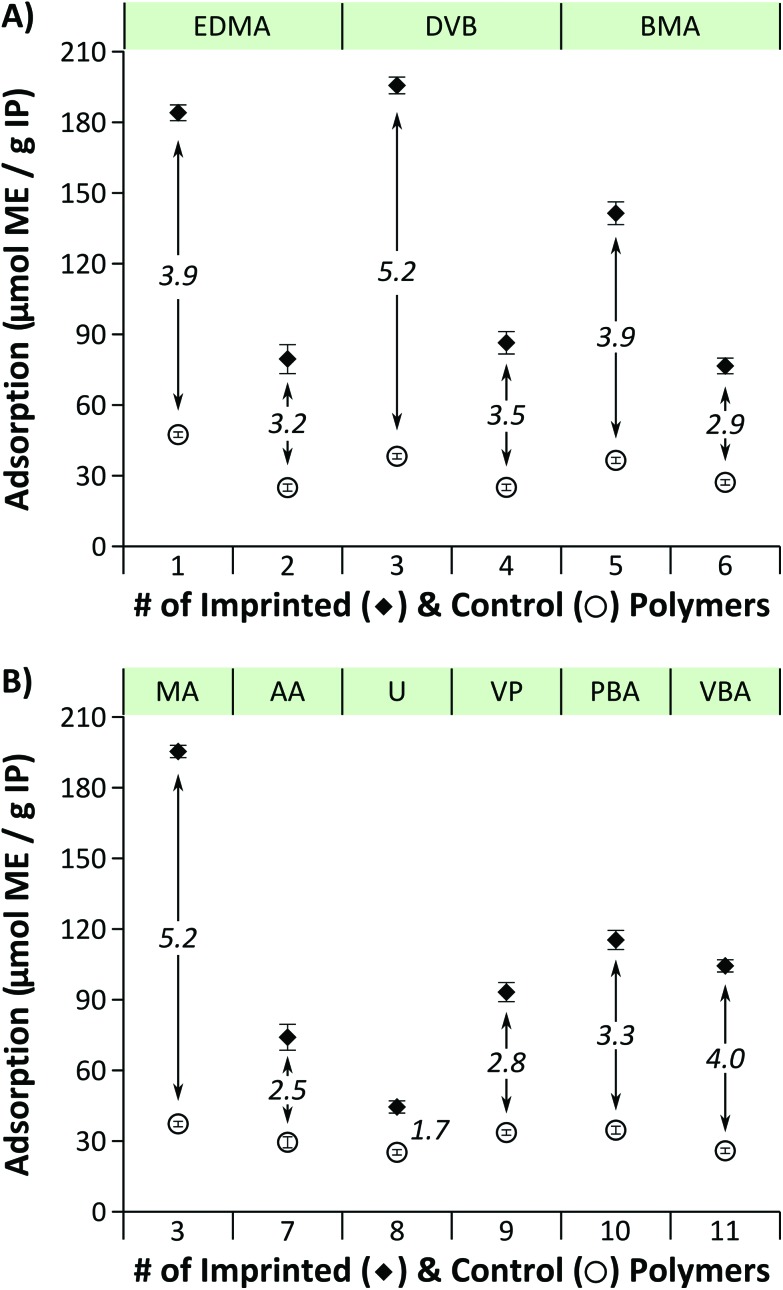
Initial adsorption of l-phenylalanine methyl ester (ME). Panel (A) includes IP1–IP6 imprinted and control polymers demonstrating the effect of crosslinker and crosslinking degree on adsorption. Panel (B) includes IP3, IP7–IP11 imprinted and control polymers showing the effect of functional monomer on adsorption. See Table 1 for the polymer compositions. Imprinting factors‡ are shown in italics.

Removal of the template after the polymerisation process was monitored by HPLC and found to be between 91% and 95%. Afterwards continuous but negligible leaching of the template was observed below the limit of detection (LOD) of the analytical method. The initial absolute adsorption values for the first adsorption–regeneration cycle are shown in Fig. 3. Duplicate assays were carried out using independently prepared polymers and the values reported are averages of the two values obtained and standard deviations were between 1 and 5 for all assays. Rejection IP1, IP3 and IP5 featuring a functional monomer to crosslinker ratio of 1/15 exhibited adsorption capacities of 185, 196 and 141 μmol ME per gram of polymer, respectively. In accordance with the expectations corresponding IP2, IP4 and IP6 polymers with a double crosslinking degree, showed about halved (57%, 56% and 46%) adsorption capacity. The imprinting factors‡
‡The retention behaviour of imprinted polymers (IP) is often compared with the reference non-imprinted, control polymer (CP), to evaluate the effect of imprinting which is often expressed by the imprinting factor defined as the ratio of the adsorbed template by an IP and its corresponding CP. were found to be between 2.9 and 5.2 proving successful incorporation of the template molecule into the polymer matrix during the polymerisation process. Polymers crosslinked at the same degree with EDMA and DVB showed similar adsorption capacities whilst BMA crosslinking at the lower crosslinking degree resulted in about 26% lower values compared to EDMA and DVB crosslinking. The highest adsorption capacity and imprinting factor was demonstrated by IP3 comprising methacrylic acid (MA) as a functional monomer and DVB as a crosslinker. The polymers prepared with the other functional monomers showed 41–77% lower adsorption capacities (Fig. 3B).

### Adsorption performance in the long term

**Fig. 4:**
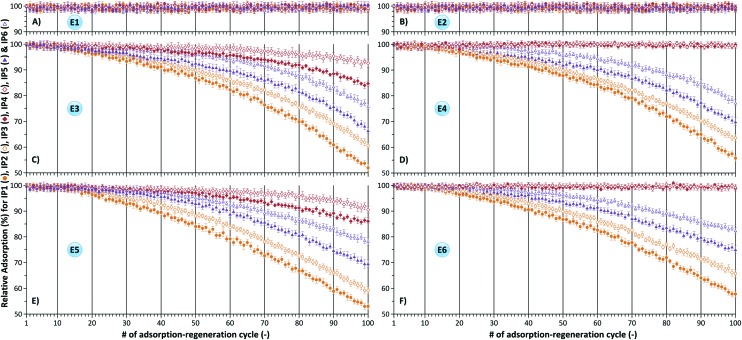
Relative adsorption of ME on IP1–6 demonstrating the effect of crosslinker and crosslinking degree on the reusability of imprinted polymers. Template extraction was carried out using methods E1 (A), E2 (B), E3 (C), E4 (D), E5 (E) and E6 (F) in accordance with Table 2.

**Fig. 5:**
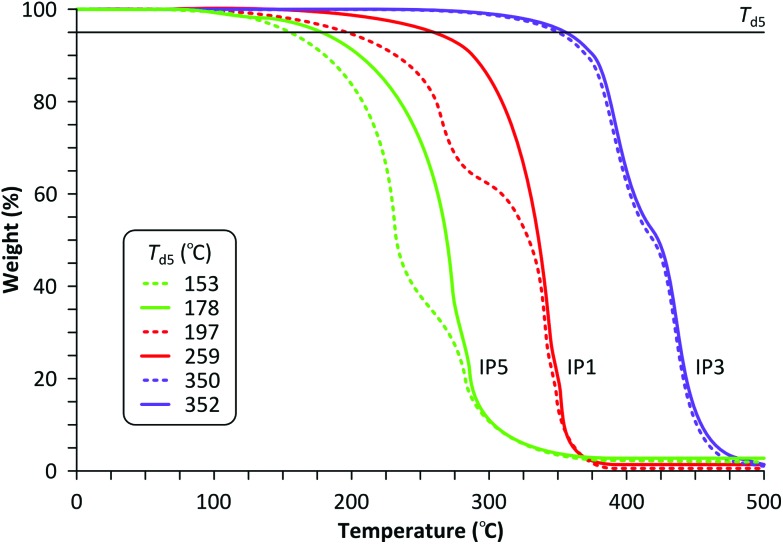
TGA profiles and 5% decomposition temperature values (*T*_d5_) for BMA, EDMA and DVB crosslinked IP5, IP1 and IP3, respectively. Solid and dotted lines show the thermal stability after the 1^st^ and 100^th^ adsorption–regeneration cycle using E6, respectively.

In order to evaluate the long-term stability and reusability of the imprinted polymers adsorption–regeneration cycles were performed up to 100 times, and the results are summarized in Fig. 4 and 5 as relative adsorption compared to the initially obtained adsorption values (see Fig. 3). Duplicate assays were carried out using independently prepared polymers and the values reported are averages of the two values obtained and standard deviations were between 1 and 3 for all assays where no polymer degradation occurred. The conditions for regeneration comprised of acidic, basic, room temperature and elevated temperature during extraction. It can be concluded that DVB crosslinking results in the most stable polymer irrespective of the degree of crosslinking. IP3 (1/15 MA/DVB) and IP4 (1/30 MA/DVB) can be reused at least 100 times without loss of affinity towards the template molecule using extraction methods E1 and E2 as shown in Fig. 4A and B, respectively. However, Fig. 4C and E reveal a relative adsorption decrease of about 5–15% using extraction methods E3 and E5, respectively (see Table 2 for extraction conditions). These acidic and basic extractions were done in methanol. In contrast, extraction methods E4 and E6 included an additional step of aqueous HCl washing resulting in no performance loss of the polymers as depicted in Fig. 4D and F, respectively. The observed high stabilities of DVB crosslinked polymers are in accordance with the fact that stationary phases based on DVB have been successfully used for many years in reversed-phase high performance liquid chromatography due to their excellent chemical, pH and temperature stability.^21^

**Fig. 6:**
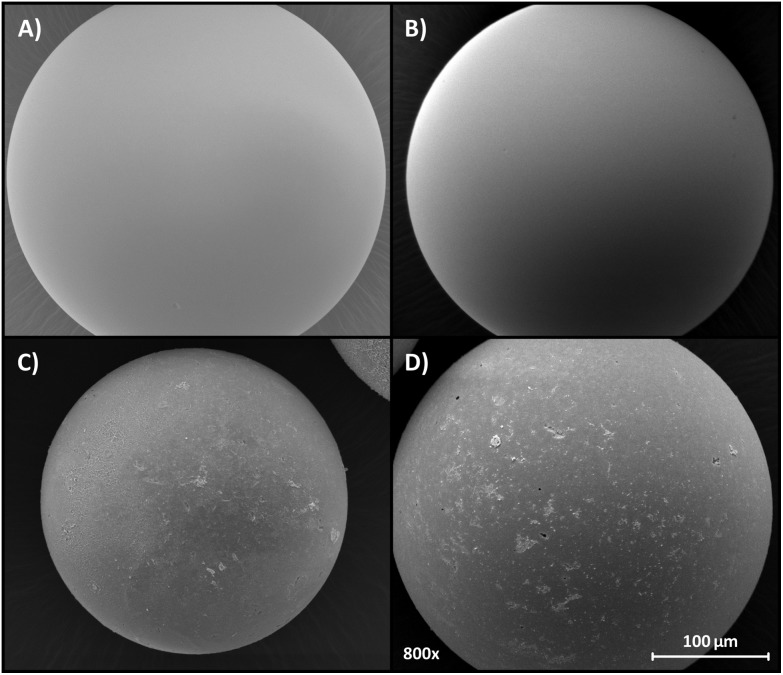
Panel A shows a typical SEM image of freshly prepared IPs. Panel B shows a typical SEM image of IP1, IP3 and IP5 microspheres after the 100^th^ cycle using the extraction method E2. Panels C and D reveal the deterioration of IP1 and IP5 after the 100^th^ cycle using the extraction method E6, respectively. See Table 2 for the regeneration methods.

In contrast, the performance of EDMA and BMA crosslinked polymers deteriorates over time under both acidic (Fig. 4C and D) and basic (Fig. 4E and F) extraction conditions. The thermal stability profiles for IP1, IP3 and IP5 with the observations on adsorbent reusability are shown in Fig. 5. Whilst the 5% decomposition temperature values (*T*_d5_) for BMA and EDMA crosslinked polymers decreased by 14% (from 178 °C to 153 °C) and 24% (from 259 °C to 197 °C), respectively, the DVB-based polymer had the highest *T*_d5_ value of 350 °C, which remained virtually the same. Fig. 6 shows how the morphology of the IP particles is affected by the regeneration method. The initial surface of all polymer particles is uniform (Fig. 6A) and remains the same even after 100 adsorption–regeneration cycles using the extraction method E2 (Fig. 6B).

**Fig. 7:**
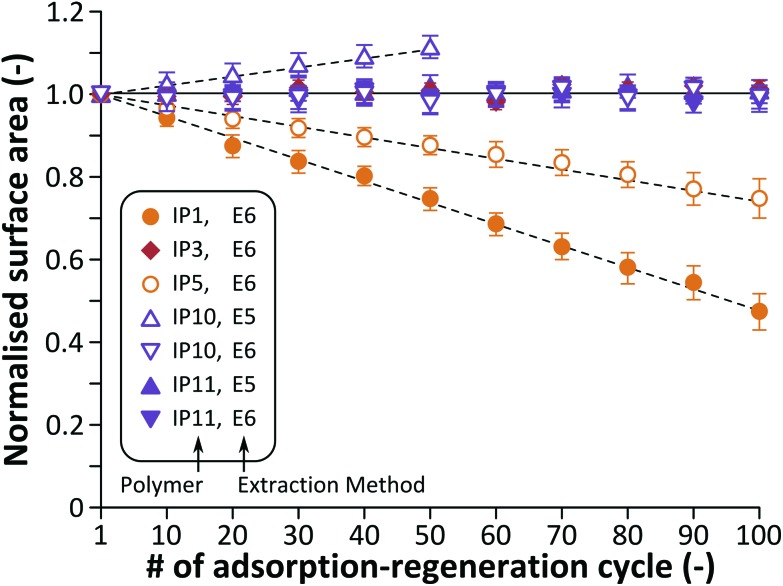
Normalised surface area obtained by Brunauer–Emmett–Teller (BET) analysis. Using the extraction method E6 the surface area for IP1 and IP5 monotonously decreases while for IP3, IP10 and IP11 remains virtually constant over time. On the other hand, the surface area increases for IP10 when the extraction method E5 is employed. See Table S2† in the ESI for the absolute surface area values.

However, the deterioration of IP1 and IP5 by the 100^th^ cycle resulted in an irregular surface with defects (Fig. 6C and D). A comparison of the surface areas for IP1, IP3 and IP5 shows the same trend (Fig. 7). The morphology of the DVB crosslinked polymer remains virtually the same over time, while the surface area for the EDMA- and BMA-based polymers monotonously decrease by 52% and 25% over 100 cycles, respectively. The decrease in surface area can be the result of the partial collapse of the 3D polymer network.

**Fig. 8:**
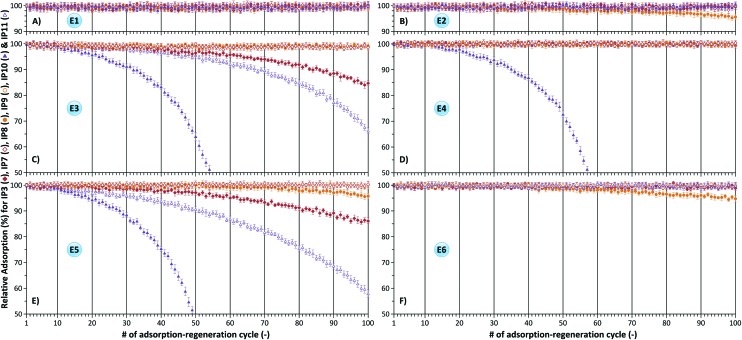
Relative adsorption of ME on IP3, IP7–11 demonstrating the effect of functional monomers on the reusability of imprinted polymers. Template extraction was carried out using methods E1 (A), E2 (B), E3 (C), E4 (D), E5 (E) and E6 (F) in accordance with Table 2.

In general the relative adsorption can decrease as much as 50% by the 100^th^ adsorption–regeneration cycle. In all assays polymers having a higher crosslinking degree demonstrated higher stability and longer reusability. For instance IP2 (1/30 MA/EDMA) demonstrated about 10% higher adsorption than IP1 (1/15 MA/EDMA) at the 100^th^ adsorption–regeneration cycle (Fig. 4C–F). Fig. 8 reveals the effect of the functional monomer on the reusability of imprinted polymers. AA and VP functional monomers in combination with the lower DVB crosslinking degree (1/15) yielded imprinted polymers (IP7 and IP9, respectively) which can be reused at least 100 times irrespective of the regeneration method (Fig. 8A–F). However, both acidic and basic conditions for template extraction using methods E3 and E5 (see Table 2) result in a performance loss of boronic acid (IP10) and carboxylic acid (IP3 and IP11) based polymers as shown in Fig. 8C and E. Nonetheless, incorporation of an aqueous HCl wash of the polymers resulted in significant improvement in performance for carboxylic acid based IP3 and IP11 (Fig. 8D and F). Both aliphatic and aromatic carboxylic acid polymers can be reused up to 100 adsorption–regeneration cycles using extraction methods E4 and E6.

On the other hand, the performance loss of boronic acid based IP10 can only be fully avoided with the extraction method E6, which requires basic conditions. An elevated temperature and basic regeneration conditions resulted in about 5% decrease in adsorption for the urea based IP8 by the 100^th^ adsorption–regeneration cycle as shown in Fig. 8B, E and F, respectively. These results are in accordance with the literature reporting that urea derivatives are more stable at acidic pH and their stability decreases by increase in temperature for all pH values.^22^

The evaluation of imprinted polymer reusability in the literature is limited to 5–10 adsorption–regeneration cycles.^23–25^ However, these short-term studies can lead to hasty conclusions. The results of short-term studies cannot be used to draw conclusions on the long-term reusability of imprinted polymers. Interpretation of the very same data presented in Fig. 4 and 8 leads to different conclusions if solely the first 10 cycles are taken into account instead of the 100 cycles. In most cases long-term performance deterioration could not have been predicted merely based on the first 10 cycles.

**Fig. 9:**
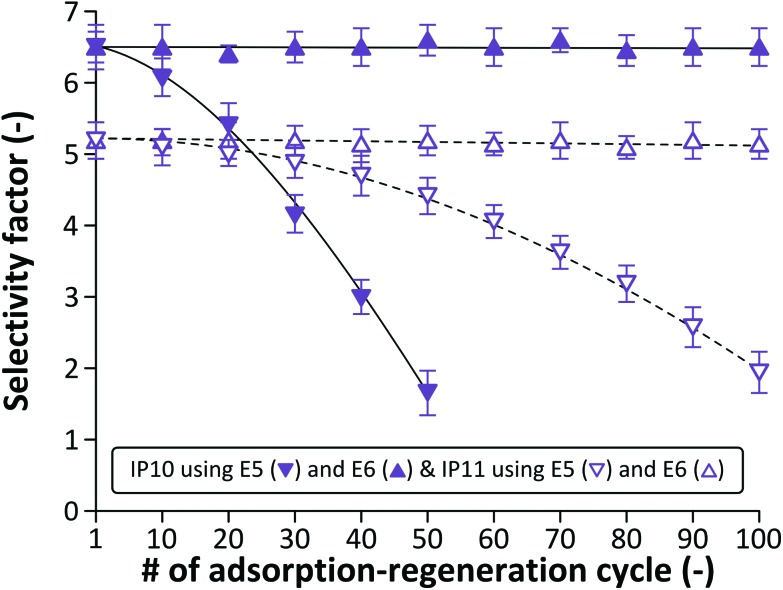
Selectivity factors for boronic acid (IP10) and carboxylic acid (IP11) based polymers using extraction methods E5 and E6. The results demonstrate that an acid wash enables maintaining the initial selectivity of the polymers.

The dummy template (BE) was used to investigate the selectivity of the polymers. Selectivity factors were calculated as the ratio of adsorbed ME and BE, and the results are summarized in Fig. 9. In order to investigate whether the selectivity deteriorates in accordance with the loss of adsorption capacity, the boronic acid (IP10) and the carboxylic acid (IP11) based polymers were tested for BE adsorption at every 10^th^ adsorption–regeneration cycle. Proving successful incorporation of the template into the polymer matrix during the polymerisation process, selectivity factors of 6.5 and 5.2 were achieved during the first cycle for IP10 and IP11, respectively. Extraction methods E5 and E6 led to decreased and maintained selectivity, respectively.

These observations are in line with the changes in the adsorption capacity shown in Fig. 8E and F. Consequently, both selectivity and adsorption capacity of boronic and carboxylic acid based imprinted polymers can be maintained up to at least 100 adsorption–regeneration cycles with an acidic wash at the end of the regeneration process. From a morphological point Fig. 7 shows that the surface area for IP10 and IP11 remains virtually constant over time when the extraction method E6 is used (see Table S2 in the ESI† for the absolute values). However, without the acidic wash the surface area significantly increases: up to 11% by the 50^th^ cycle and 18% by the 100^th^ cycle. The increase in surface area is unexpected and it is probably due to the molecular level degradation of the polymer without collapse of the polymer network.

Leaching of the template and unreacted monomers from IPs is an often observed phenomenon, which is considered as one of the main drawbacks of the technology.^15^ They should be exhaustively removed in order to ensure reliable results, in particular if IPs are used for quantitative analytical applications or product purification. Such clean-up is time-consuming and requires substantial amounts of the extraction solvent. Eppler *et al.* developed an extraction device specifically designed to facilitate the rapid and efficient clean-up of imprinted polymer matrices offering significant advantages: the ability to use solvent mixtures, temperature-controlled extraction conditions, improved kinetics, and continuous control of the extraction process.^16^ However, both reversible and irreversible degradation of the polymer network can occur under basic and acidic conditions.

**Fig. 10:**
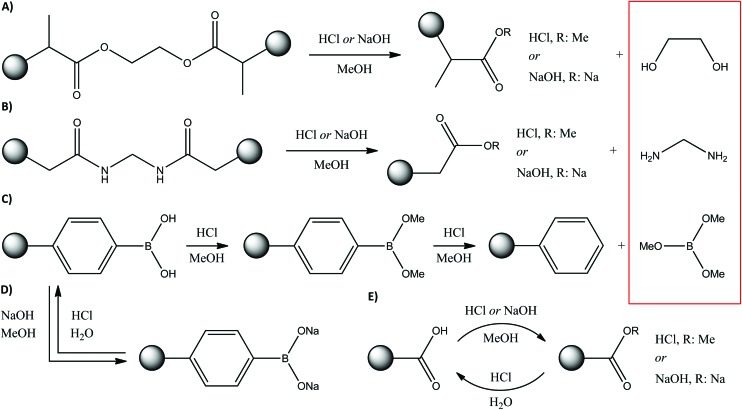
Degradation schemes of EDMA (A), BMA (B) crosslinked polymers, and esterification of acid based functional monomers *via* hydrolysis in methanol (C–E). The resulting esters or sodium salts remain part of the polymer network whilst ethylene glycol (A), methylenediamine (B) and trimethyl borate (C) leach out from the adsorbents (see in the box). The elimination by-products were isolated and their characterisation can be found in the ESI.†

EDMA and BMA crosslinked polymers can readily undergo transesterification and hydrolysis under acidic and basic conditions, respectively (Fig. 10A and B). Both reactions result in losing their crosslinking function, which eventually leads to reduced stability and reusability as demonstrated *via* TGA, SEM, adsorption and selectivity studies. Although the resulting esters or sodium salts remain part of the polymer network, the ethylene glycol and methylenediamine leach out from the adsorbents and could have an adverse effect during the application of the imprinted polymer. Boronic acids can also undergo irreversible esterification in methanol in the presence of acids and the reaction can proceed further resulting in the elimination of trimethyl borate (Fig. 10C), which again leaches out from the adsorbent. On the other hand, esterification of boronic acids is not catalysed by bases and consequently only sodium salt formation can occur, which is reversible (Fig. 10D). Such behaviour of boronic acids is the reason why extraction methods E3 and E5 result in performance loss of IP10 with regard to adsorption capacity (Fig. 8C and E) and selectivity (Fig. 9), whilst the method E6 incorporating a final acidic wash facilitates maintaining the adsorption (Fig. 8F) and selectivity performance over 100 cycles. Both the esterification and sodium salt formation of carboxylic acids are reversible (Fig. 10E), and thus the performance of IP3 and IP11 can be maintained by methods E4 and E6 (Fig. 8D and F). The decomposition mechanism presented here is in line with the observations on the surface area change over time (Fig. 7). Partial degradation of the crosslinked structure (Fig. 10A and B) results in the collapse of the polymer network decreasing the surface area, while the surface areas for the DVB-based polymer remain virtually constant.

On the other hand, the surface area for polymers with boronic acid functional groups increases over cycles when the extraction method E5 is employed resulting in trimethyl borate elimination (Fig. 10C). The DVB crosslinked polymer network is not affected by the extraction, but the binding sites change, which ultimately results in the increase of the surface area without collapse of the 3D polymer network.

It has been demonstrated that apart from template and monomer leaching, polymer degradation can also result in contamination of samples during the application of IPs. Consequently, both the monomers and the conditions for adsorbent regeneration need to be carefully selected.

## Conclusions

The reusability of imprinted polymers has a crucial role in developing applications that are reliable, economic and sustainable. The effect of crosslinkers, functional monomers and conditions for template extraction was systematically investigated to reveal the long-term stability and reusability of imprinted polymers. Adsorption–regeneration cycles were carried out 100 times for l-phenylalanine methyl ester imprinted polymers. Irrespective of the degree of crosslinking, divinylbenzene-based polymers demonstrated the most robust behaviour compared to methacrylate- and acrylamide-based polymers. These polymers can be reused at least 100 times without loss of affinity towards the template molecule under acidic and basic conditions, and an elevated temperature (65 °C). In contrast, the performance of methacrylate- and acrylamide-based polymers deteriorates over time under both acidic and basic extraction conditions. It has been demonstrated that besides the well-known template and monomer leaching, polymer degradation products can also result in contamination of samples during the application of the adsorbents. Performance loss of imprinted polymers having carboxylic acid functional moieties can be recovered by an aqueous acidic wash at the end of the regeneration cycle. However, methacrylate- and acrylamide-based polymers irreversibly degrade under both acidic and basic conditions, which results in decreased degree of crosslinking. Imprinted polymers having a boronic acid functionality should not be regenerated under acidic conditions in alcohol as it results in their irreversible degradation and elimination of trialkyl borates. Further challenges to be resolved include the regeneration of macromolecularly imprinted polymers.^26^
