# Supplementary material for: Long-term stability and reusability of molecularly imprinted polymers
Source: Polym Chem. 2016 Nov 24;8(4):666–73. doi: 10.1039/c6py01853j (PMC5361172; doi:10.1039/c6py01853j)
Supplement: Supplementary file 1 [file PY-008-C6PY01853J-s001.pdf]

Electronic Supplementary Material (ESI) for Polymer Chemistry.
This journal is © The Royal Society of Chemistry 2016

Electronic Supplementary Information

for

Long-term stability and reusability of molecularly imprinted polymers

Jozsef Kupai,[1,2] Mayamin Razali,[1] Sibel Buyuktiryaki,[3] Rustem Kecili,[3] Gyorgy Szekely[1,*]

[1]*School of Chemical Engineering & Analytical Science, The University of Manchester, The Mill, Sackville street, Manchester, M13 9PL, United Kingdom*
[2]*Department of Organic Chemistry & Technology, Budapest University of Technology & Economics, Szent Gellert ter 4, Budapest, 1117, Hungary*
[3]*Yunus Emre Vocational School, Anadolu University, Eskisehir, 26470, Turkey*

[*]Corresponding author, e-mail: gyorgy.szekely@manchester.ac.uk; Tel: +44 (0)161 306 4366

Table of contents

Figure S1.	^{1}H-NMR spectrum of 1-(4-vinylphenyl)-3-(3,5-bis(trifluoromethyl)phenyl)urea functional monomer.	2
Figure S2.	^{13}C-NMR spectrum of 1-(4-vinylphenyl)-3-(3,5-bis(trifluoromethyl)phenyl)urea functional monomer.	2
Figure S3.	^{1}H-NMR spectrum of methylenediamine dihydrochloride.	3
Figure S4.	^{13}C-NMR spectrum of methylenediamine dihydrochloride.	4
Figure S5.	^{15}N-NMR spectrum of methylenediamine dihydrochloride.	5
Figure S6.	^{1}H-NMR spectrum of trimethyl borate.	6
Figure S7.	^{13}C-NMR spectrum of trimethyl borate.	7
Figure S8.	^{11}B-NMR spectrum of trimethyl borate.	8
Figure S9.	^{1}H-NMR spectrum of ethylene glycol.	9
Figure S10.	^{13}C-NMR spectrum of ethylene glycol.	10
Table S1.	Elemental analysis of the imprinted and control polymers after template extration.	11
Table S2.	Change in BET surface area of the polymers.	11

1-(4-vinylphenyl)-3-(3,5-bis(trifluoromethyl)phenyl)urea

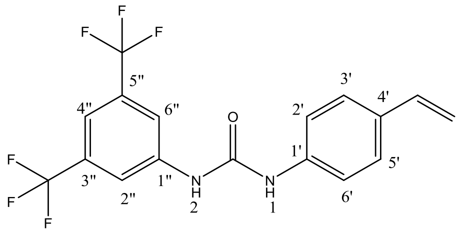

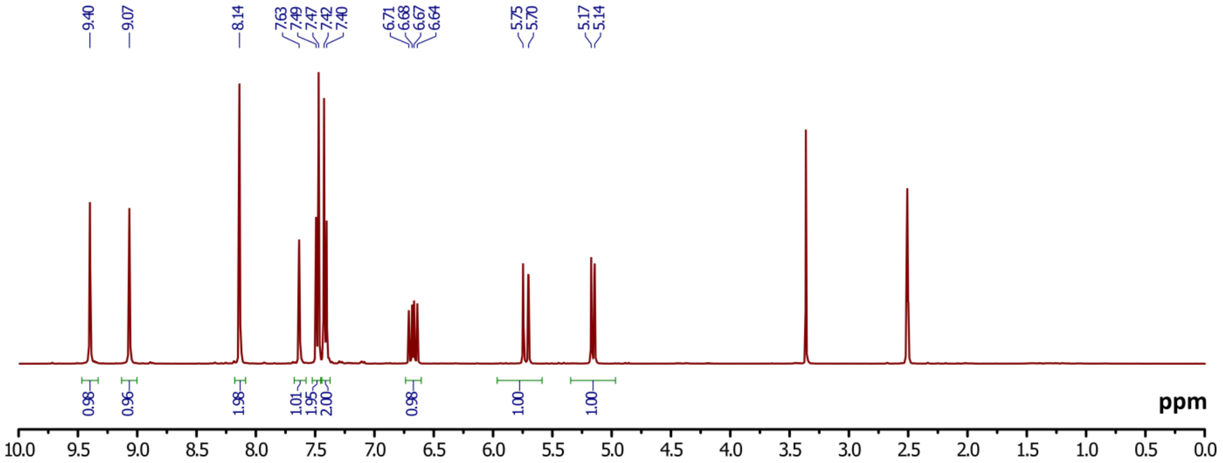

Figure S1. ^{1}H-NMR spectrum of 1-(4-vinylphenyl)-3-(3,5-bis(trifluoromethyl)phenyl)urea functional monomer. ^{1}H-NMR (400 MHz, DMSO-d_6) δ: 5.16 (1H, dd, *J*=10.8 Hz, 0.8 Hz, CH=CH_2), 5.73 (1H, dd, *J*=17.6 Hz, 0.8 Hz, CH=CH_2), 6.68 (1H, dd, J_{AB}=10.8 Hz, C*H*=CH_2), 7.41 and 7.48 (2×2H, dd, $J_{AA'BB'}$=8.6 Hz, Ar-CH-2',6' and Ar-CH-3',5'), 7.63 (1H, s, Ar-CH-4"), 8.14 (2H, s, Ar-CH-2", 6"), 9.07 (1H, s, urea-NH-3), 9.40 (1H, s, urea-NH-1) ppm.

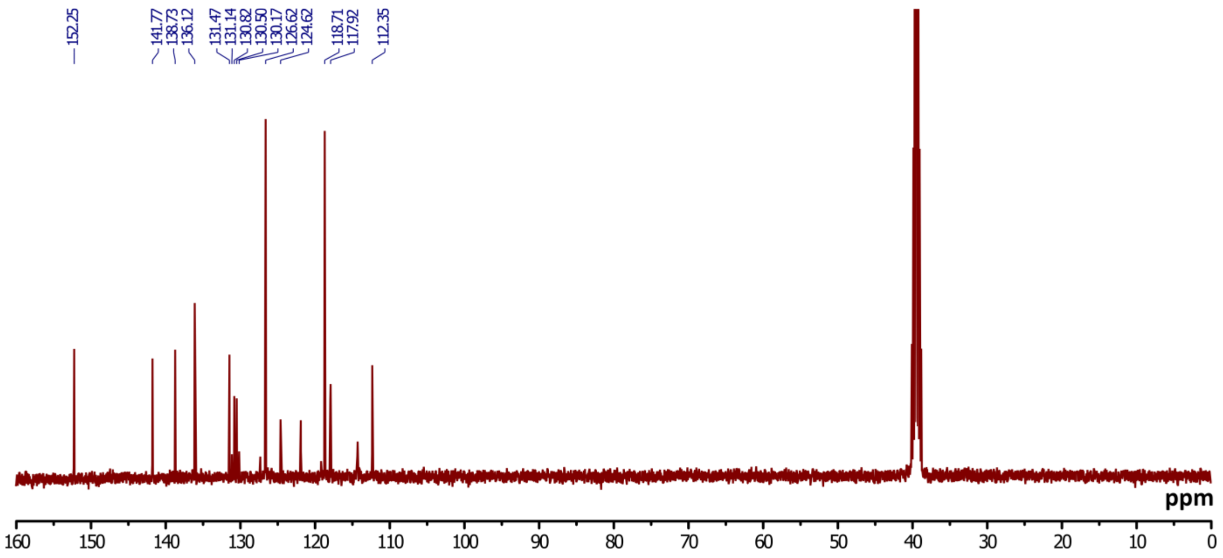

Figure S2. ^{13}C-NMR spectrum of 1-(4-vinylphenyl)-3-(3,5-bis(trifluoromethyl)phenyl)urea functional monomer. ^{13}C-NMR (100 MHz, DMSO-d_6) δ: 112.35 (CH=CH_2), 117.92 (Ar-*C*H-4"), 118.71 (Ar-CH-2", 6"), 124.62 (CF_3), 126.62 (Ar-CH-3',5'), 127.33 (Ar-*C*H-4'), 130.66 (q, Ar-CH-3",5"), 131.47 (Ar-CH-2', 6'), 136.12 (*C*H=CH_2), 138.73 (Ar-CH-1'), 141.77 (Ar-CH-1"), 152.25 (urea C) ppm.

Methylene diamine dihydrochloride

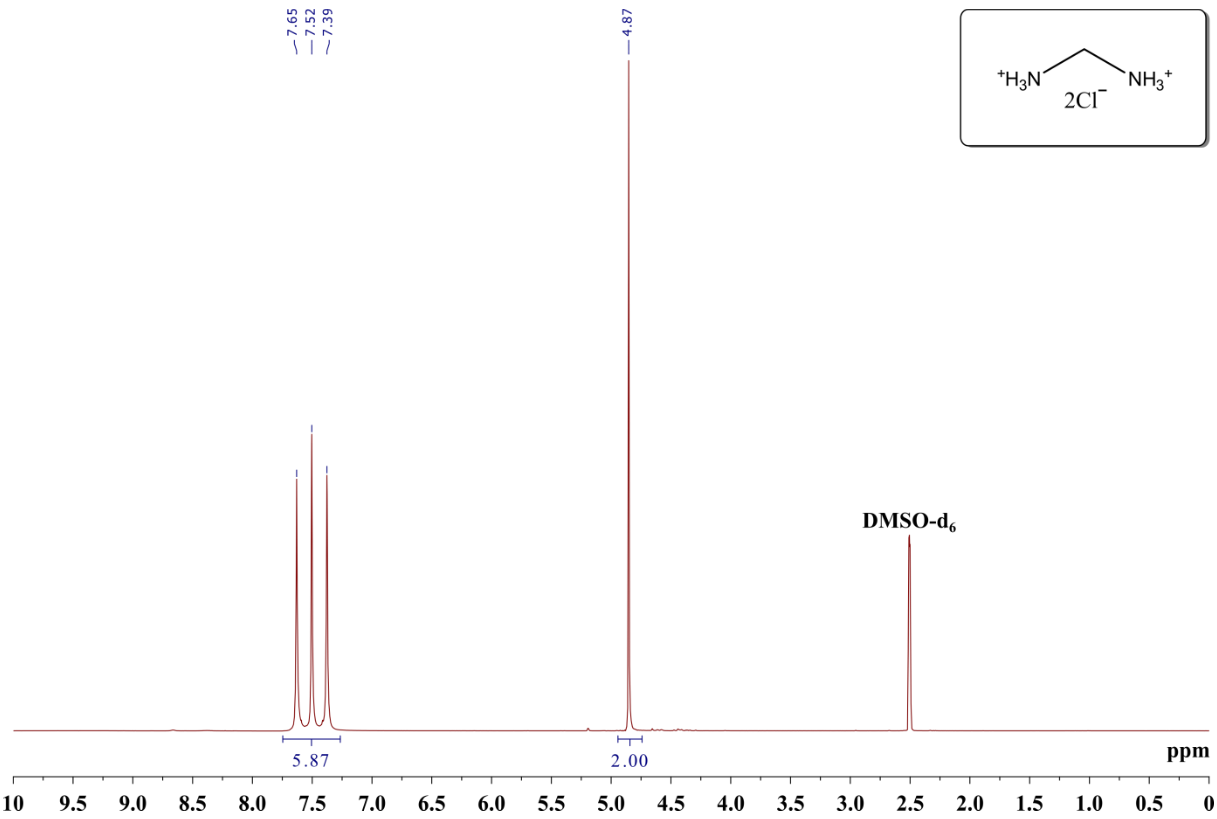

Figure S3. ^{1}H-NMR spectrum of methylenediamine dihydrochloride. ^{1}H-NMR (400 MHz, DMSO-d_6) δ: 4.87 (2H, s, CH_2), 7.52 (6H, t, *J*=50.8 Hz, NH_3^+) ppm.

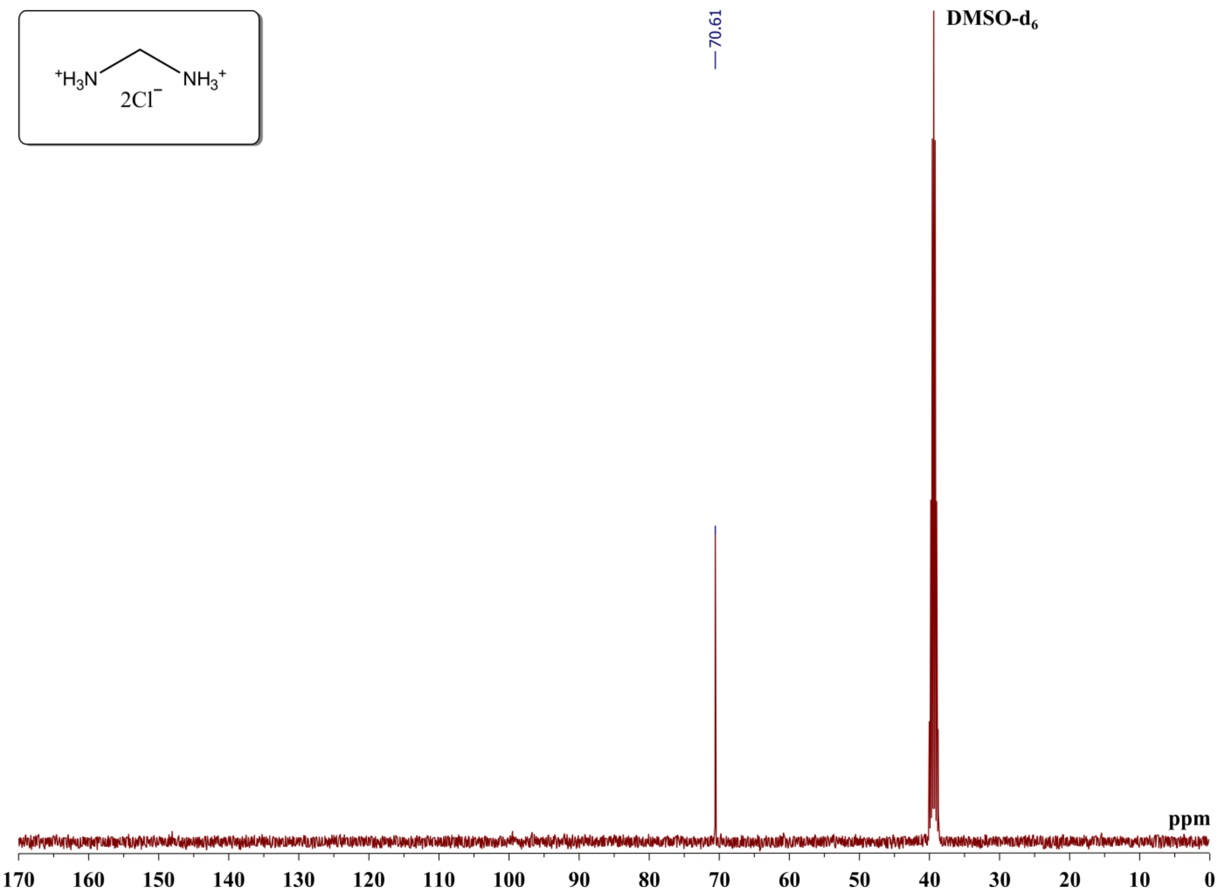

Figure S4. ^{13}C-NMR spectrum of methylenediamine dihydrochloride. ^{13}C-NMR (100 MHz, DMSO-d_6) δ: 70.61 (CH_2) ppm.

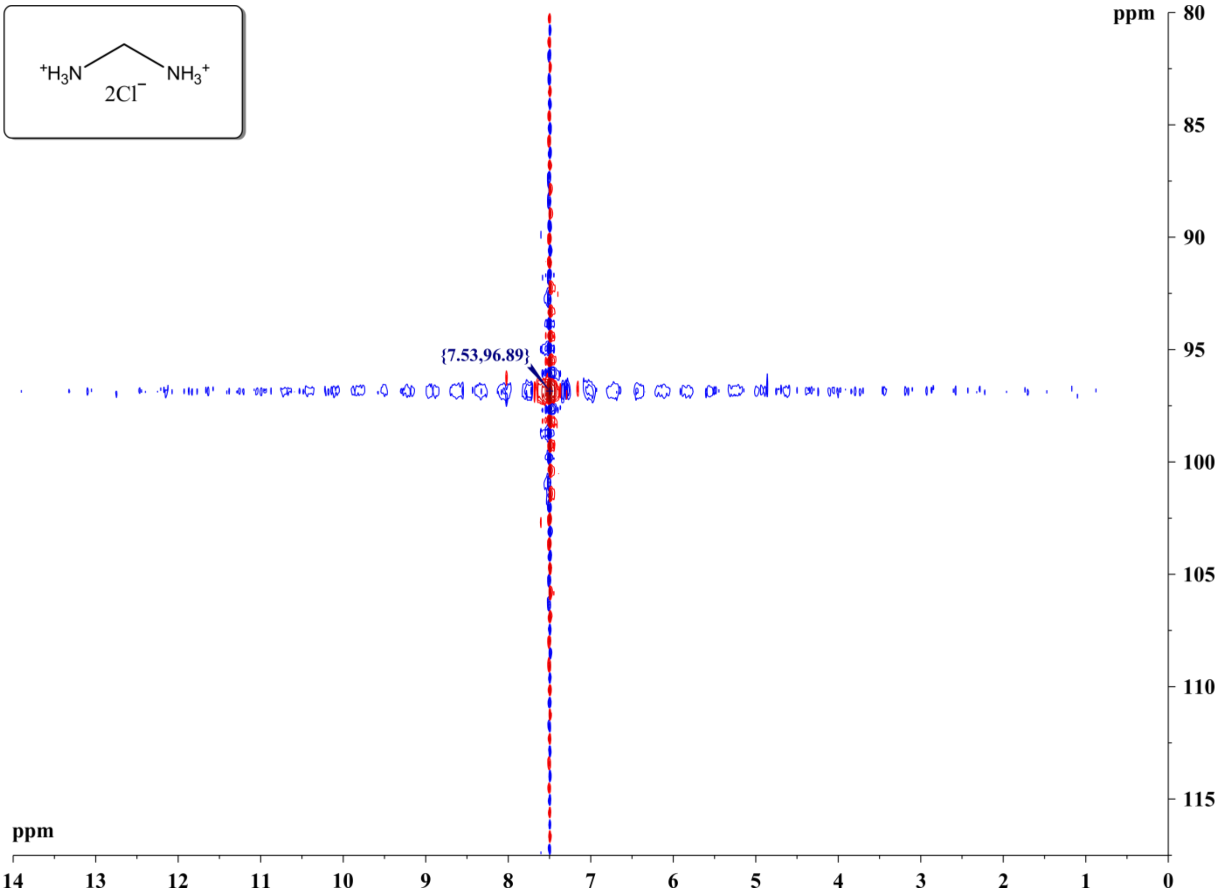

Figure S5. ^{15}N-NMR spectrum of methylenediamine dihydrochloride. ^{15}N-NMR (50.8 MHz, DMSO-d_6) δ: 96.85 (NH_3^+) ppm.

Trimethyl borate

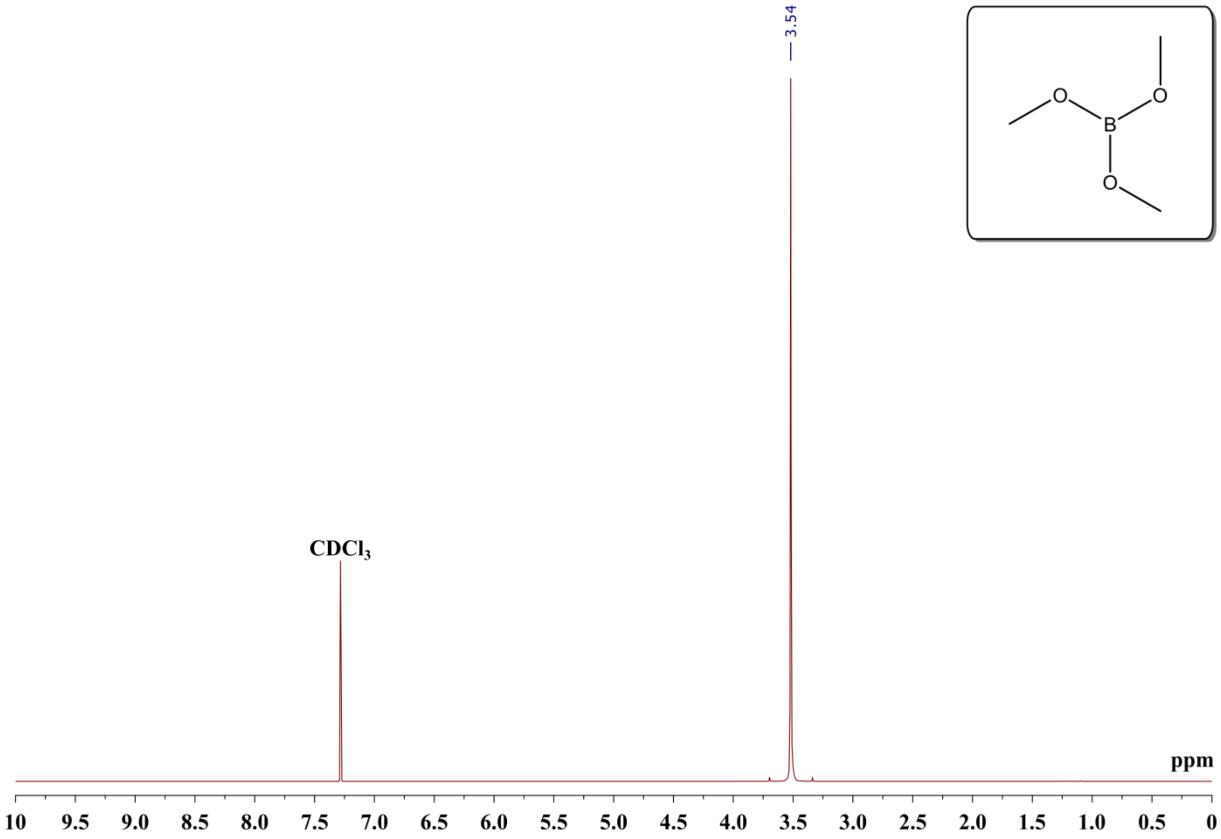

Figure S6. ^{1}H-NMR spectrum of trimethyl borate. ^{1}H-NMR (400 MHz, $CDCl_3$) δ: 3.52 (9H, s, OCH_3) ppm.

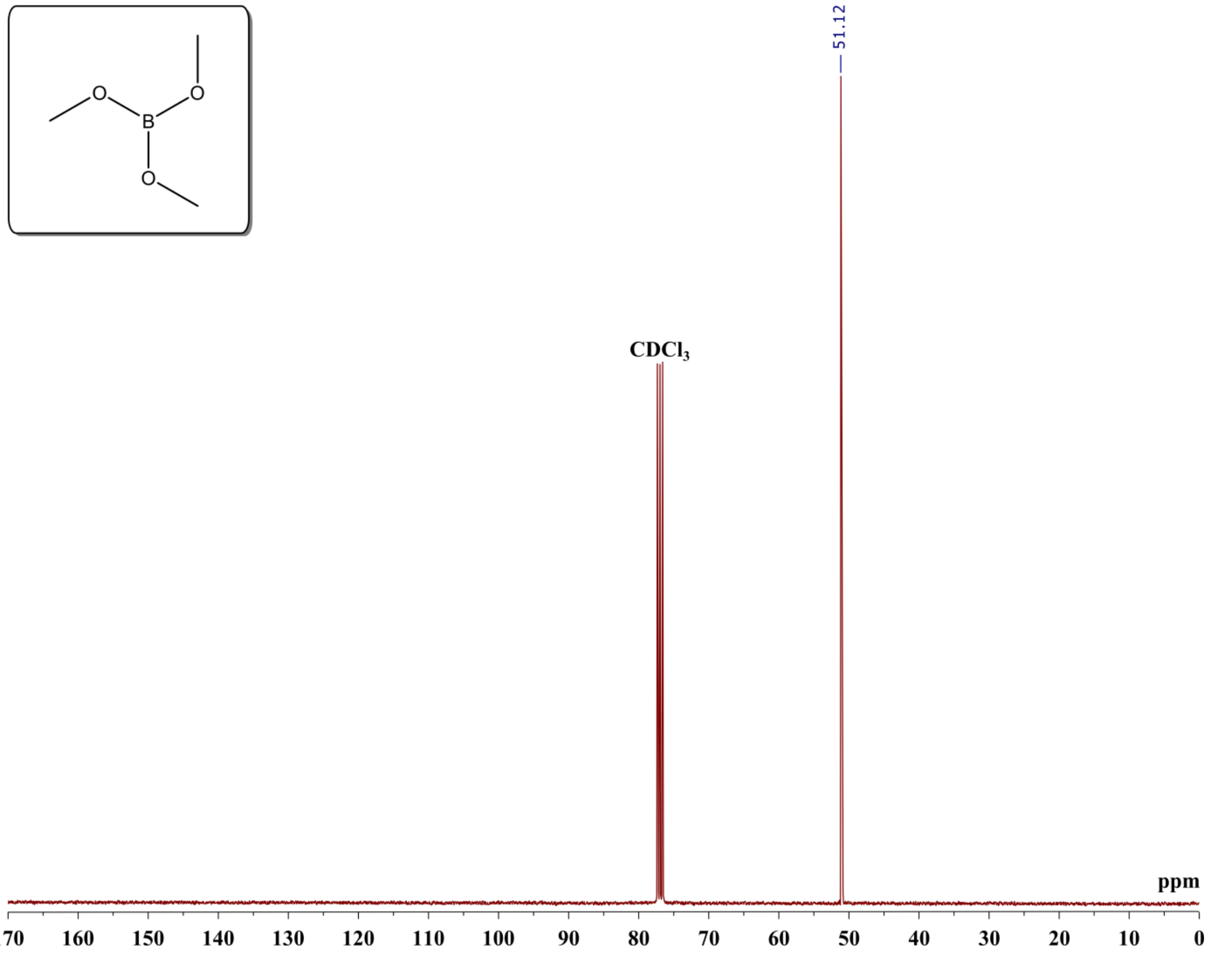

Figure S7. ^{13}C-NMR spectrum of trimethyl borate. ^{13}C-NMR (100 MHz, $CDCl_3$) δ= 51.17 (OCH_3) ppm.

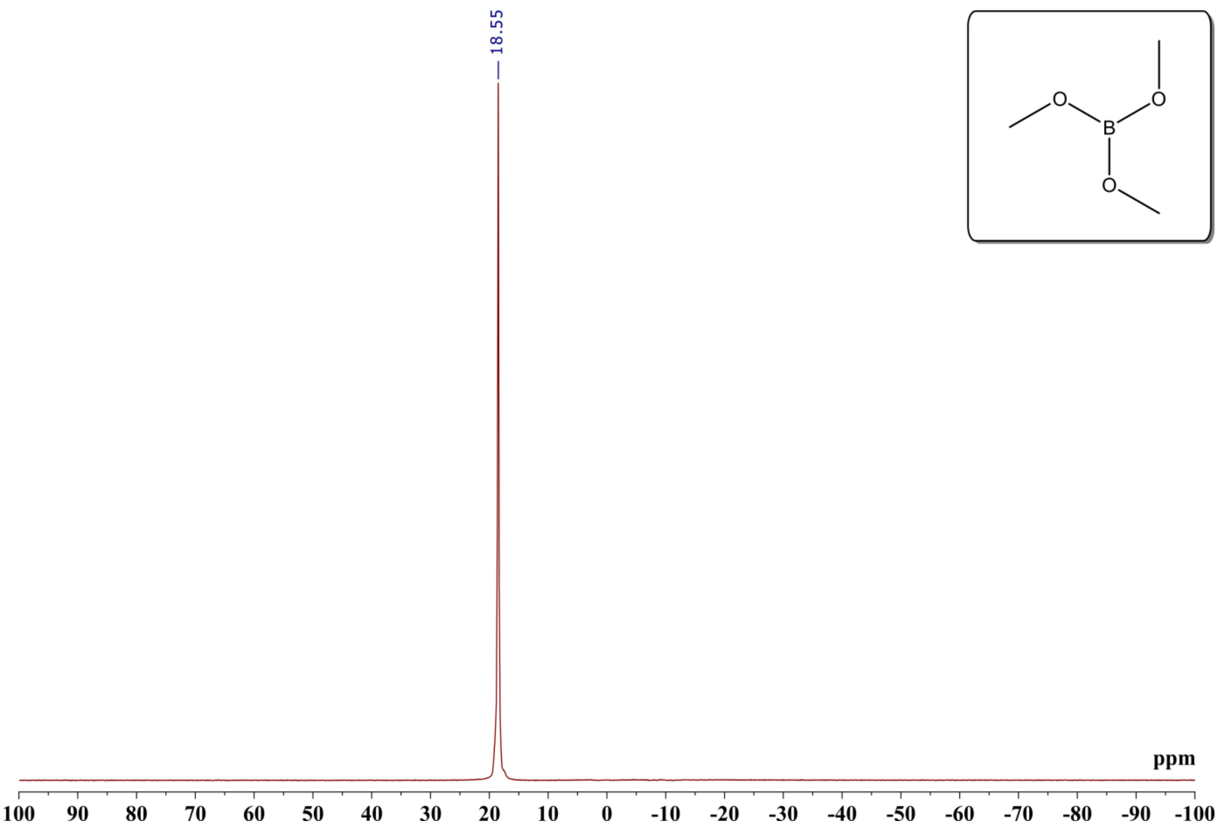

Figure S8. ^{11}B-NMR spectrum of trimethyl borate. ^{11}B-NMR (160.5 MHz, $CDCl_3$) δ= 18.49 [$B(OCH_3)_3$] ppm.

Ethylene glycol

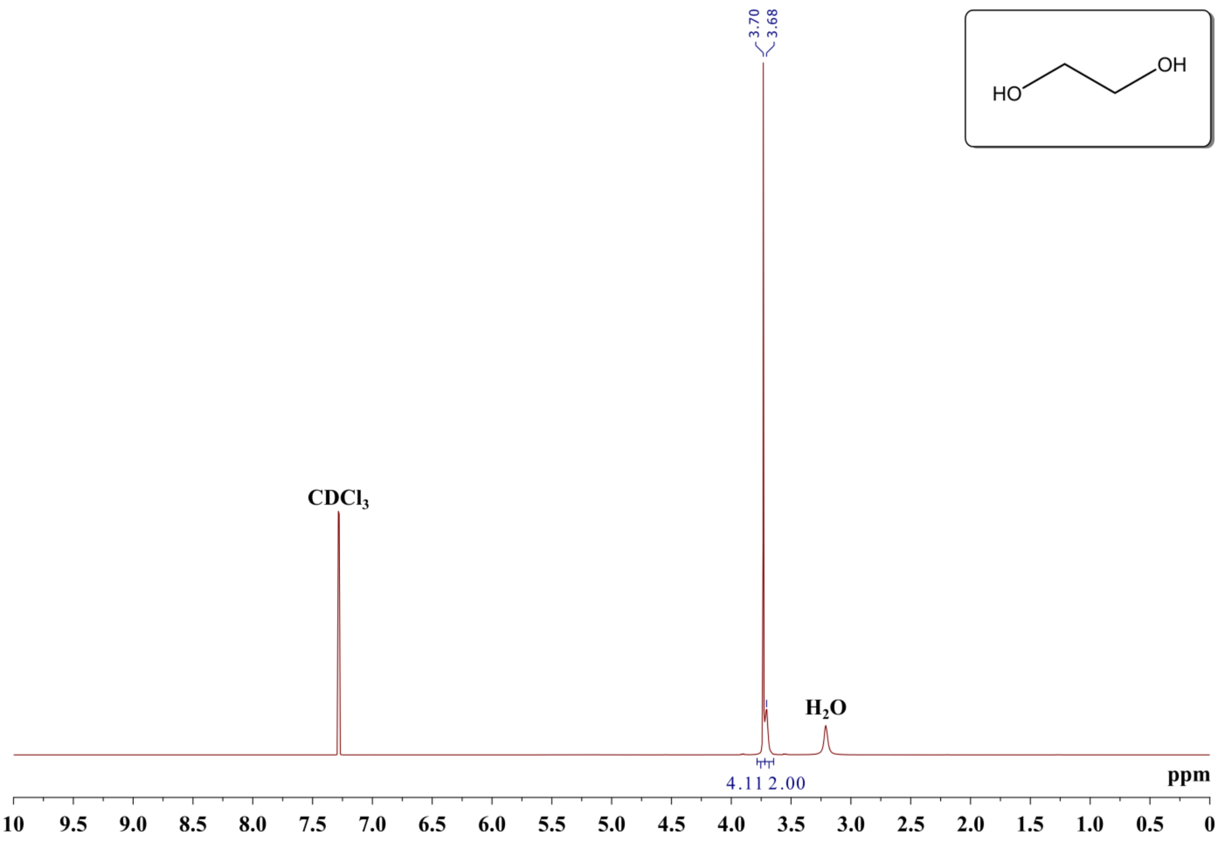

Figure S9. ^{1}H-NMR spectrum of ethylene glycol. ^{1}H-NMR (400 MHz, $CDCl_3$) δ: 3.68 (2H, br. s, OH), 3.70 (4H, s, CH_2) ppm.

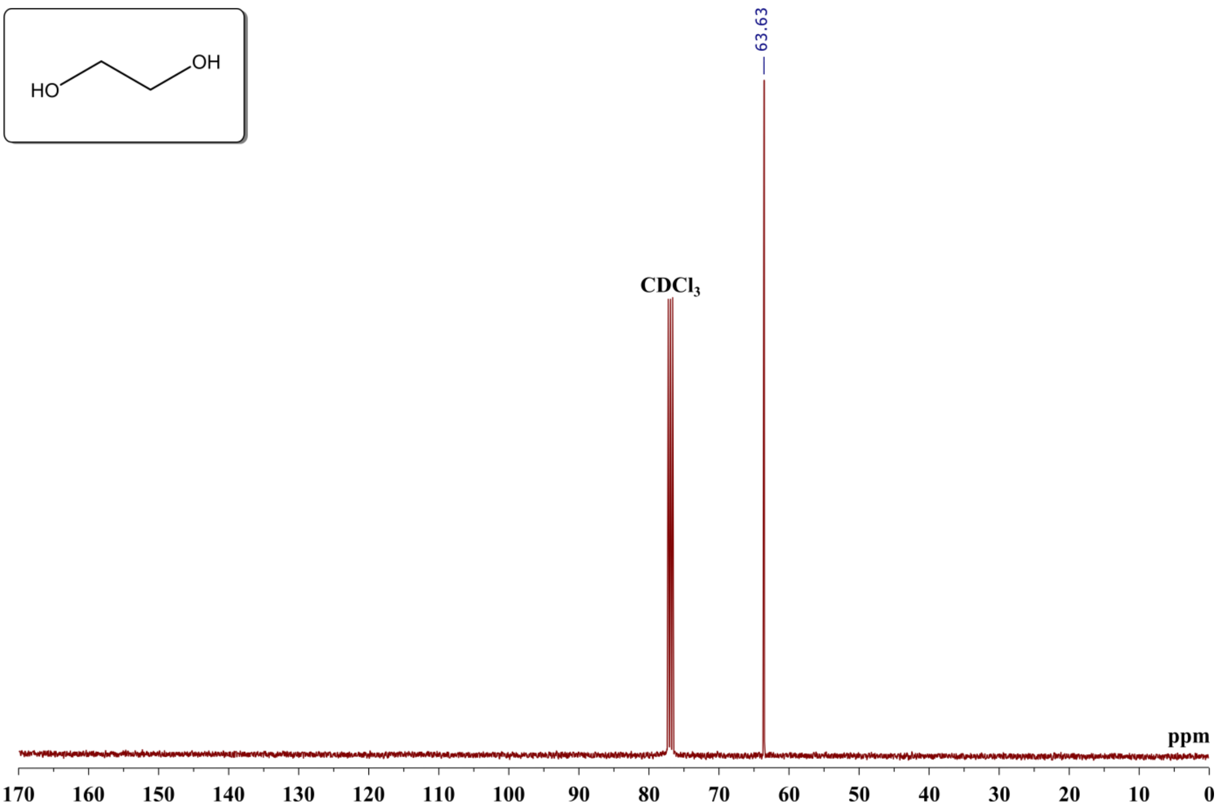

Figure S10. ^{13}C-NMR spectrum of ethylene glycol. ^{13}C-NMR (100 MHz, $CDCl_3$) δ: 63.67 (CH_2) ppm.

Table S1. Elemental analysis of the imprinted and control polymers after template extration. Anal. Calcd/Found. See Table 1 in the main manuscript for the conditions of polymer preparation.

**Polymers**	**Carbon** (%)	**Hydrogen** (%)	**Nitrogen** (%)
IP1	60.87/60.83	7.24/7.25	0.43/0.41
IP2	60.59/60.52	7.17/7.19	0.23/0.26
IP3	61.04/60.96	7.22/7.22	0.02/0.05
IP4	60.74/60.78	7.14/7.16	0.24/0.22
IP5	61.27/61.25	7.19/7.23	0.02/0.04
IP6	60.95/60.86	7.16/7.18	0.01/0.05
IP7	60.87/60.82	7.24/7.21	0.43/0.45
IP8	60.58/60.63	7.21/7.25	0.43/0.39
IP9	61.04/60.11	7.22/7.24	0.02/0.05
IP10	60.88/60.92	7.16/7.12	0.46/0.51
IP11	61.27/61.34	7.19/7.20	0.02/0.03
CP1	60.87/60.89	7.24/7.28	0.43/0.45
CP2	60.59/60.48	7.17/7.15	0.23/0.19
CP3	61.04/60.96	7.22/7.25	0.02/0.05
CP4	60.74/60.78	7.14/7.11	0.24/0.22
CP5	61.27/61.35	7.19/7.18	0.02/0.04
CP6	60.95/61.21	7.16/7.13	0.01/0.00
CP7	60.87/60.59	7.24/7.27	0.43/0.45
CP8	60.58/60.51	7.21/7.18	0.43/0.41
CP9	61.04/60.87	7.22/7.20	0.02/0.05
CP10	60.88/60.96	7.16/7.16	0.46/0.42
CP11	61.27/61.34	7.19/7.22	0.02/0.04

Table S2. Change in BET surface area of the polymers given in $m^2.g^{-1}$ unit. The template extraction method is given after the forward slash. See Table 1 and 2 in the main manuscript for the polymer compositions and the conditions of the template extratcion methods, respectively.

# of adsorption-regeneration cycle	IP1/#6	IP3/#6	IP5/#6	IP10/#6	IP10/#5	IP11/#6	IP11/#5
1	62±2	64±3	60±1	50±2	51±1	48±2	49±1
10	58±2	64±2	58±1	50±2	52±1	48±2	49±2
20	54±2	64±3	56±1	50±2	53±1	48±2	51±2
30	52±2	65±2	55±1	50±2	54±1	48±2	52±1
40	50±2	64±2	54±1	49±2	55±1	48±2	53±1
50	47±2	64±2	52±2	49±2	56±1	48±1	55±2
60	43±2	63±2	51±2	50±2	-	48±1	55±1
70	39±3	65±2	50±3	50±2	-	48±2	56±1
80	36±4	64±2	48±3	50±2	-	48±1	57±1
90	34±4	65±2	46±3	50±2	-	47±2	57±1
100	29±4	64±2	44±4	49±2	-	47±2	58±1
